# Cluster analysis of long COVID symptoms for deciphering a syndrome and its long-term consequence

**DOI:** 10.1007/s12026-024-09465-w

**Published:** 2024-04-16

**Authors:** J. Niewolik, M. Mikuteit, S. Klawitter, D. Schröder, A. Stölting, K. Vahldiek, S. Heinemann, F. Müller, GMN. Behrens, F. Klawonn, A. Dopfer-Jablonka, S. Steffens

**Affiliations:** 1 Department of Rheumatology and Immunology, Hannover Medical School, Carl-Neuberg-Str. 1, 30625 Hannover, Germany; 2 Dean’s Office — Curriculum Development, Hannover Medical School, Hannover, Germany; 3 Institute for Information Engineering, Ostfalia University of Applied Sciences, Wolfenbüttel, Germany; 4 Department of General Practice, University Medical Center Göttingen, Göttingen, Germany; 5 German Center for Infection Research (DZIF), Partner Site Hannover, Brunswick, Germany; 6 Helmholtz Centre for Infection Research, Brunswick, Germany

**Keywords:** Long COVID symptoms, Cluster analysis, COVID-19

## Abstract

The long-term symptoms of COVID-19 are the subject of public and scientific discussions. Understanding how those long COVID symptoms co-occur in clusters of syndromes may indicate the pathogenic mechanisms of long COVID. Our study objective was to cluster the different long COVID symptoms. We included persons who had a COVID-19 and assessed long-term symptoms (at least 4 weeks after first symptoms). Hierarchical clustering was applied to the symptoms as well as to the participants based on the Euclidean distance *h* of the log-values of the answers on symptom severity. The distribution of clusters within our cohort is shown in a heat map.

From September 2021 to November 2023, 2371 persons with persisting long COVID symptoms participated in the study. Self-assessed long COVID symptoms were assigned to three symptom clusters. Cluster A unites rheumatological and neurological symptoms, cluster B includes neuro-psychological symptoms together with cardiorespiratory symptoms, and a third cluster C shows an association of general infection signs, dermatological and otology symptoms. A high proportion of the participants (*n* = 1424) showed symptoms of all three clusters. Clustering of long COVID symptoms reveals similarities to the symptomatology of already described syndromes such as the Myalgic Encephalomyelitis/Chronic Fatigue Syndrome (ME/CFS) or rheumatological autoinflammatory diseases. Further research may identify serological parameters or clinical risk factors associated with the shown clusters and might improve our understanding of long COVID as a systemic disease. Furthermore, multimodal treatments can be developed and scaled for symptom clusters and associated impairments.

## Introduction

Since the worldwide spread of the novel Coronavirus Disease 2019 (COVID-19) to a pandemic in the beginning of 2020, not only the acute infection and its mortality but also the long-term consequences have become subject of public and scientific interest [1]. A proportion of COVID-19 survivors, both hospitalized and non-hospitalized, have persisting symptoms for more than 30, 60, or even 90 days after primary infection, referred to as “long haul COVID,” post-acute sequelae of COVID-19 (PASC) or long COVID [2]. The World Health Organization (WHO) has published a definition of the post COVID-19 condition specifying the condition with persisting symptoms as fatigue, shortness of breath, and cognitive dysfunction lasting for at least 2 months [3]. This definition is to be sharpened by further research and possibly may include several long COVID syndromes. When investigating long-term symptoms more than 28 days after a severe acute respiratory syndrome coronavirus 2 (SARS-CoV-2) infection several studies, reviews, and meta-analyses show fatigue as the most frequent and strongly presenting symptom. In addition, dyspnea, impaired concentration, and arthromyalgia are also regularly mentioned symptoms, depending mostly on the time after infection [2, 4, 5].

Cluster analysis as an exploratory data analysis method has been used in several studies to investigate the co-prevalence of symptoms. Using this approach to characterizing post-viral syndromes associated with COVID-19 can help to discover similarities to previously described syndromes and to derive novel hypothesis pathogenetic pathways and possible treatment approaches. Furthermore, our results may inform health services researchers on how to design multimodal treatment regimens that specifically address clusters instead of single symptoms and thus lead to an improvement of patient care. Previous cluster analysis studies focus on symptoms of acute COVID-19 or also psychosomatic symptoms associated with the pandemic situation [6, 7]. Conducted cluster analysis on long COVID symptoms differ methodologically in the included participants, symptom selection, and method to cluster the symptoms [8]. Kenny et al. have identified three distinct long COVID clusters in a cohort of 233 individuals: Cluster A with pain-related symptoms, cluster B with cardiorespiratory symptoms, and cluster C characterized by a significantly lower number or reported symptoms per individual [9]. Goldhaber et al. used exploratory factor analysis to identify five clusters among 16 symptoms in a study comprising 999 participants [10]: (1) Gastrointestinal, (2) neurocognitive, (3) musculoskeletal, (4) airway, and (5) cardiopulmonary symptoms without overlapping clusters. Tsuchida et al. performed a hierarchical cluster analysis and also identified five clusters among 23 symptoms including 500 participants [11].

The aim of this study is to identify symptom clusters in people with long COVID and to describe their prevalence. We use a cross-sectional data sample of a large long COVID online survey to cluster self-reported symptoms.

## Methods

### Recruitment and eligibility criteria

Participants were informed about the study via press articles, social media, doctors’ offices, and the distribution of leaflets in high-traffic locations in the municipalities of the northern German state of Lower Saxony (population 7.9 million). Lower Saxony is a large federal state with both urban and rural areas. The age distribution, income distribution, and the proportion of migrants are comparable to that of the Federal Republic of Germany. We also passed on information about the study to self-help groups throughout Germany. Participants could enroll their selves on our project websites. Before enrollment, the participants needed to digitally declare consent to participate. As the symptoms of long COVID differ between minors and adults [12, 13], we only included people aged 18 and over.

### Questionnaire and grouping of participants

An online survey created and pre-tested by an interdisciplinary team was accessed via direct link or through registration on the project website http://www.defeat-corona.de. The survey was developed in German language using SoSci Survey (SoSci Survey GmbH, München, Germany). The collected data was pseudonymized and stored on servers of Hannover Medical School.

The survey contained questions about demographic baseline characteristics, prior SARS-CoV-2 infection and vaccination, as well as a self-assessment questionnaire about the occurrence of several common symptoms [14]. Twenty-seven symptoms across different organ systems were queried: general (e.g., fever, loss of appetite), musculoskeletal (e.g., muscle pain, joint pain), cardiopulmonary (e.g., dyspnea, tachycardia), neurology (e.g., fatigue/brain fog, dizziness, loss of smell and taste). Symptom strength could be graded on an 11-point Likert scale ranging between 0 = no symptoms and 10 = strongest symptoms. Symptoms were chosen based on the WHO definition of long COVID symptoms and the guidelines of the UK National Institute for Health and Care Excellence (NICE) [3, 14].

A prior SARS-CoV-2 infection was defined either by a self-assessed positive polymerase chain reaction (PCR) test, positive antibody test (anti-Spike/anti-nucleocapsid protein immunoglobulin G), or positive rapid antigen test. Long COVID symptoms were defined as persisting or new symptoms more than 4 weeks after infection with SARS-CoV-2. This long COVID definition was based on the German Public Health Institute (Robert Koch-Institut) and the NICE [15, 16].

### Statistical analysis

Pseudonymized data was stored using Microsoft Excel 2019, version 2010 (Microsoft Corporation, Redmond, WA, USA) and password secured on the internal server of Hanover Medical School. Duplicates were excluded from statistical analysis and participants that stated to have first COVID-19 symptoms before January 2020 as the first confirmed COVID-19 cases in Germany were in January 2020. Patient and symptom characteristics were described as proportions with percentages or as means with standard deviation or median with interquartile range (IQR).

The results on symptoms and clusters were calculated with weighted data according to the gender ratio (62.5% women and 37.5% men) of the long COVID diagnoses (ICD-10-GM version 2021 U09/U09.9) based on data from a German health insurance company. The weighting was achieved by oversampling. For symptom frequency, symptom severity was classified in a dichotomous system. Therefore, the cutoff for counting a symptom as present was calculated as the median for each symptom. When the strength of a symptom was above the median, this was counted as present. The mean strength of the symptoms was thus calculated as the mean of all participants who rated this symptom over the cutoff.

For symptom clustering, individual symptom strength on the Likert scale was used as a continuous variable. Hierarchical clustering was applied to the symptoms as well as to the participants based on the Euclidean distance *h* of the log-values of the answers on the Likert scale. The Lance-Williams dissimilarity update formula was used to join three clusters [17]. The Lance-Williams formula was chosen to promote the formation of clusters with minimal increase in variance upon merging. The number of three clusters was chosen according to clinical relevance. A heat map with dendrograms for the clustering results for the symptoms and the participants was generated. Clustering and statistical analysis was carried out with the open source software R, version 4.1.2 (packages stats (4.2.0), gplots (3.1.3), fmsb (0.7.3)) [18].

## Results

### Description of cohort

From September 2021 to November 2023, 2371 persons completed the questionnaire who reported persisting symptoms more than 4 weeks after an infection with SARS-CoV-2. Hereafter, we refer to these persons as “long COVID participants.” After using the oversampling technique to weight the dataset by gender, our dataset consisted of 3046 people. Table 1 shows detailed participant characteristics of our unweighted sample and the weighted sample. The following data refers only to the weighted sample.

**Table 1:** Demographics of survey respondents

Variable/data	Long COVID patients (weighted)	Long COVID patients (unweighted)
Number of participants (*n*)	2371	3046
Time from beginning of the infection to survey (weeks) mean (SD)	41.3 (28)	41.9 (28)
EQ-5D VAS mean (SD)	52.4 (22.6)	52.7 (22.9)
Gender, *n* (%)		
Female	1904 (80.3)	1904 (62.5)
Male	451 (19.0)	1142 (37.5)
Diverse	9 (0.4)	0 (0)
Not answered	7 (0.3)	0 (0)
Age (years) mean (SD)	43.7 (12.4)	43.8 (12.6)
Hospitalization, *n* (%)	195 (8.2)	
Yes, regular ward	149 (6.3)	200 (6.6)
Yes, intensive care unit	35 (1.5)	75 (2.5)
Irregular	11 (0.5)	12 (0.4)
Comorbidities, *n* (%)		
No	822 (34.7)	1136 (37.3)
Yes	1491 (62.9)	1839 (60.4)
Not answered	58 (2.4)	71 (2.3)
Cardiovascular^a^, *n* (%)	434 (18.3)	571 (18.8)
Metabolic^b^, *n* (%)	627 (26.4)	731 (24)
Pneumological^c^, *n* (%)	318 (13.4)	380 (12.5)
Inflammatory and autoimmune^d^, *n* (%)	773 (32.6)	950 (31.2)
Renal failure, *n* (%)	18 (0.8)	25 (0.8)
Neuropsychiatric^e^, *n* (%)	492 (20.8)	574 (18.8)
Chronic pain, *n* (%)	140 (5.9)	173 (5.7)
Solid cancer, *n* (%)	11 (0.5)	15 (0.5)
Sars-CoV-2 test*, *n* (%)		
PCR Test positive	2156 (90.9)	2759 (90.6)
Anti-Body Test positive	218 (9.2)	288 (9.5)
Antigen Rapid Test positive	317 (13.4)	418 (13.7)

*Multiple answers possible

^a^Cardiovascular: hypertension, heart failure, coronary heart disease, atrial fibrillation/flutter, other cardiac arrhythmias, peripheral occlusive disease of the extremities

^b^Metabolic: diabetes mellitus type 2, gout, obesity, cholelithiasis, thyroid disease

^c^Pneumological: bronchial asthma, chronic obstructive pulmonary disease

^d^Inflammatory and autoimmune: Chronic hepatitis, HIV, Crohn’s disease/ulcerative colitis, psoriasis, allergies, neurodermatitis, rheumatism, polymyalgia/polymyalgia rheumatica, other autoimmune diseases

^e^Neuropsychiatric: migraine, epilepsy/seizure disorder, Parkinson’s disease, dementia, schizophrenia/mania, depression

### Symptoms and symptom frequency

**Fig. 1:**
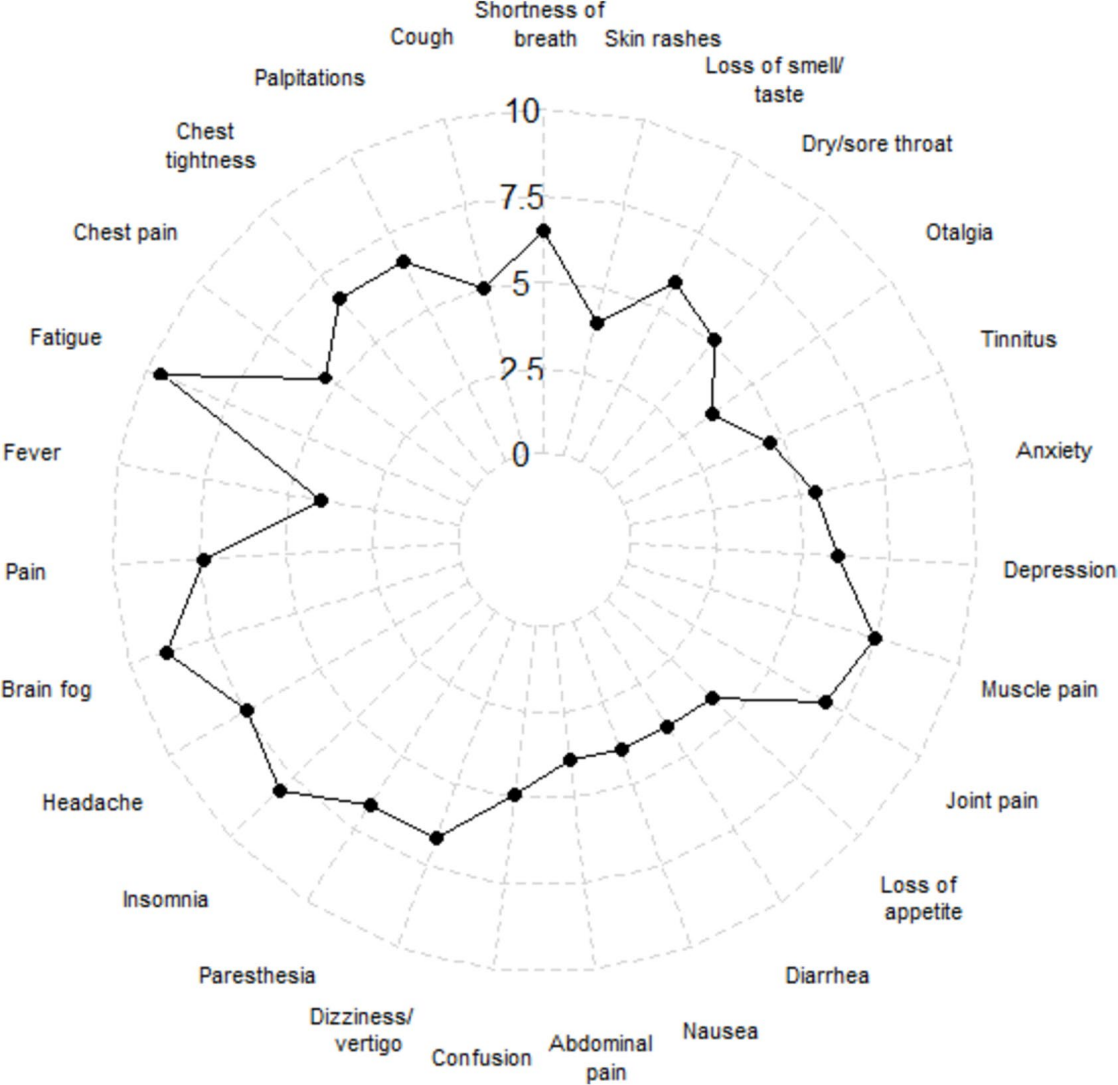
Mean strength of long COVID symptoms. Radar Chart — mean of strength is plotted for each symptom after applying the cutoff with a strength of 0/10 in the middle to a strength of 10/10 on the outside

After using the individual median as a cutoff, the most frequent symptoms were chest pain (49.3%), dizziness/vertigo (49.1%), and respiratory symptoms (48.2% with shortness of breath and 48.1% with cough). The two symptoms rated as the most severe were fatigue and brain fog both with mean severity of 9.6 and 8.9 on the 10-point Likert-Scale. The least-mentioned symptoms in the long COVID participants were otalgia (23.2%) and skin rashes (25.2%). Figure 1 shows the mean strength of each symptom illustrated in a radar chart. Table 2 shows detailed information on the frequency and mean strength of all symptoms.

**Table 2:** Self-reported symptoms (level of symptoms ranging from 0 to 10 of participants with symptoms and long COVID, *n* = 3,046 after weighting for gender using oversampling)

Symptoms	*n* (%)	Mean (SD)
Fever	971 (31.9)	4.1 ± 2.8
Pain	1378 (45.2)	7.3 ± 1.6
Diarrhea	1078 (35.4)	4 ± 2.6
Muscle pain	1397 (45.9)	7.4 ± 1.6
Joint pain	1385 (45.5)	6.8 ± 1.9
Skin rashes	769 (25.2)	4 ± 2.8
Loss of appetite	1203 (39.5)	4.2 ± 2.7
Abdominal pain	1097 (36)	3.8 ± 2.5
Shortness of breath	1469 (48.2)	5.6 ± 1.7
Cough	1465 (48.1)	5.1 ± 2.4
Palpitations	1451 (47.6)	6.5 ± 1.9
Chest tightness	1362 (44.7)	6.7 ± 1.7
Chest pain	1503 (49.3)	5.4 ± 2.4
Fatigue	1039 (34.1)	9.6 ± 0.5
Brain fog	1183 (38.8)	8.9 ± 0.8
Headache	1440 (47.3)	7.4 ± 1.6
Insomnia	1379 (45.3)	8 ± 1.4
Paresthesia	1358 (44.6)	6.7 ± 2.2
Dizziness/vertigo	1497 (49.1)	6.1 ± 2.2
Confusion	1334 (43.8)	4.9 ± 2.4
Depression	1266 (41.6)	6.6 ± 1.8
Anxiety	1451 (47.6)	5.4 ± 2.6
Nausea	1181 (38.8)	3.9 ± 2.6
Tinnitus	1111 (36.5)	4.5 ± 2.9
Otalgia	707 (23.2)	3.5 ± 2.5
Dry/sore throat	1381 (45.3)	5.1 ± 2.5
Loss of smell/taste	1406 (46.2)	5.9 ± 3.4

Cutoff for a symptom was calculated as the median for each symptom. When the strength of a symptom was above the median, this was counted as a mention

### Cluster analysis

**Fig. 2:**
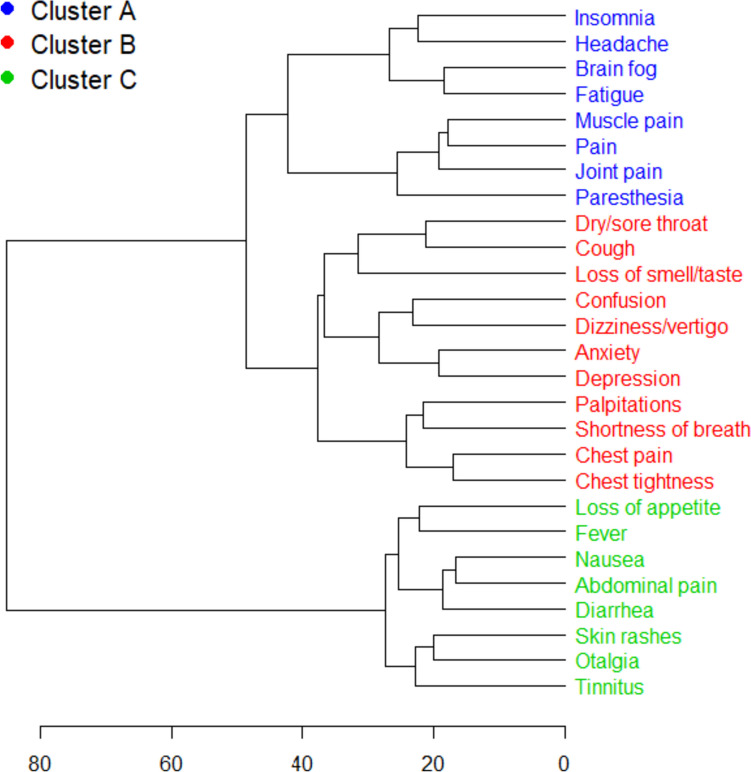
Dendrogram of long COVID symptom clusters. Symptoms were clustered according to heaped joint occurrence and strength in *n* = 3046 long COVID participants (weighted for gender using oversampling). The x-axis shows the value of the distance metrics. The smaller the distance-value, the closer the clusters. Three big clusters can be described. Cluster A unites rheumatologic and neurologic/neuro-psychosomatic symptoms. Cluster B: neurological and psychological symptoms occur frequently together with cardiologic and pulmonary symptoms. Cluster C: co-occurrence of gastrointestinal symptoms with tinnitus/otalgia, skin rashes, and infection-associated symptoms

Long-term symptoms persisting more than 4 weeks after the SARS-CoV-2 infection among long COVID participants can be assigned to three symptom clusters (Fig. 2). The symptoms insomnia, headache, brain fog, fatigue, muscle pain, pain and joint pain cluster, and paresthesia together in cluster A (distance *h* = 42.5). This cluster seems to unite rheumatologic and neurological symptoms. Cluster B shows that neurological (confusion, dizziness/vertigo, loss of smell and taste) and psychological (anxiety, depression) symptoms occur frequently together with cardiologic and pulmonary symptoms (*h* = 37.7). In cluster C, interestingly, we found a co-occurrence of gastrointestinal symptoms with tinnitus/otalgia, skin rashes, and infection-associated symptoms such as fever and loss of appetite (*h* = 27.4).

### Cluster distribution on patients

**Fig. 3:**
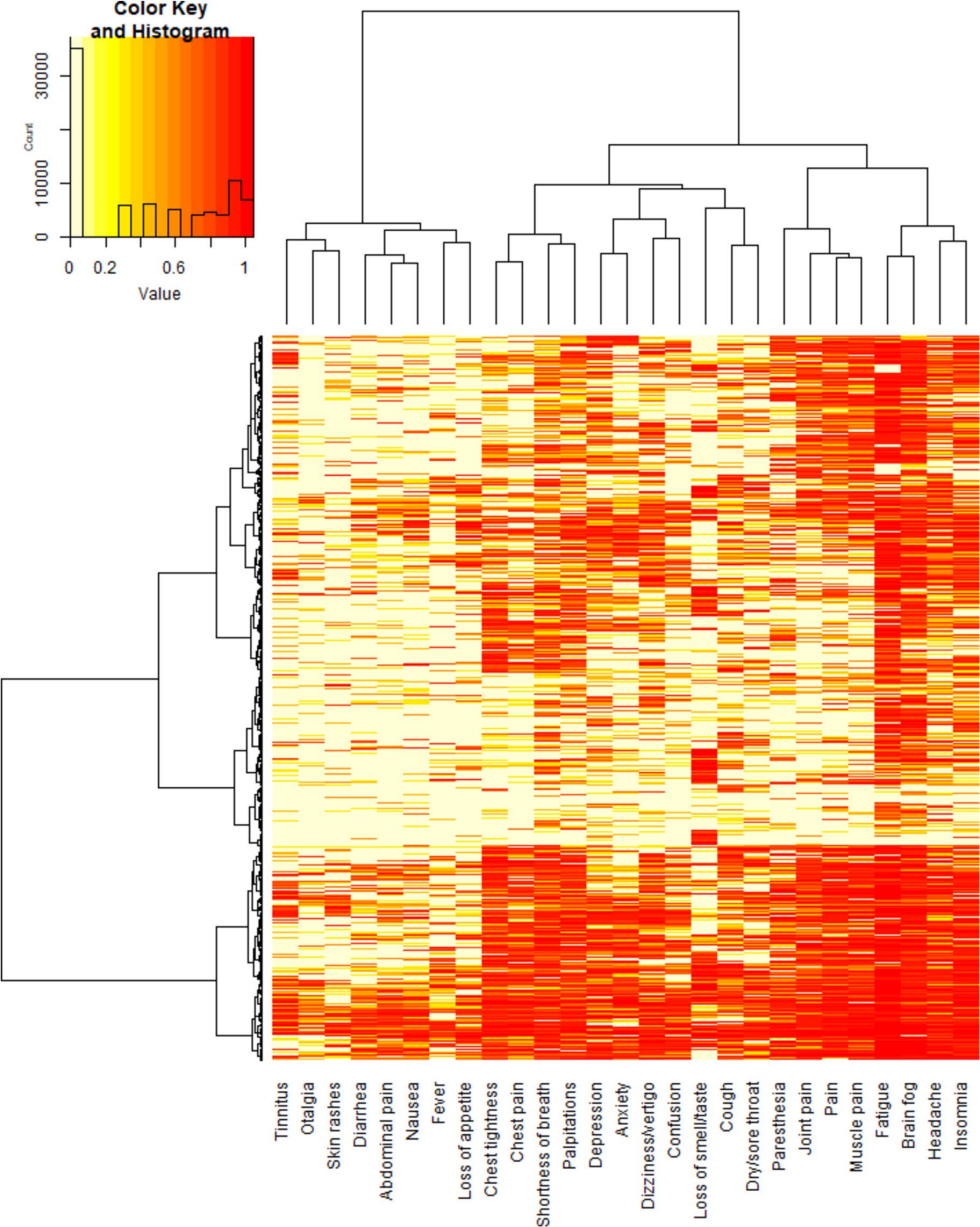
Long COVID and long COVID patients clustered in a heat map. Both symptoms (x-axis) and participants (y-axis) were clustered. Colors show the strength of each symptom with red = 10/10 and white = 0/10, *n* = 3046 (weighted for gender using oversampling)

In the heat map, both the participants and symptoms were clustered (Fig. 3) and the distribution of the clusters A, B, and C among within our participants is visible. At the top of the heat map, *n* = 1424 patients are clustered who show symptoms of all three clusters. At the bottom of Fig. 3, *n* = 899 participants are clustered, who mostly show symptoms of clusters A and B.

## Discussion

In our cohort of mostly non-hospitalized COVID-19 survivors, we could sharpen the description of long-term symptoms by clustering them in three symptom clusters. Fatigue and brain fog were the most frequent long-term symptoms after a SARS-CoV-2 infection, confirming results from other studies [5, 19]. In a recent meta-analysis regarding 18 follow-up studies on long COVID symptoms, among the most prevalent symptoms were fatigue and weakness with a pooled prevalence of 28%, cognitive impairments (19%), depression symptoms (23%), and dyspnea (18%) [5]. Our study shows a similar ranking of symptoms with respiratory and neurological symptoms being the most frequent. Despite a similar ranking of the participants symptoms with long COVID show a higher prevalence of most symptoms than described in the literature. This is especially interesting as our cutoff for present symptom already uses the rather conservative median of each individual symptom strength. The higher frequency could have its origin in the self-reported assessment of symptoms. Even when using a rather conservative cutoff to determine symptom prevalence we could show that long COVID symptoms are not only prevalent after hospitalized COVID-19 but also after “mild” acute disease.

In a meta-analysis on long COVID symptoms, 13 of 18 studies only examined hospitalized patients [5]. In a recently published observational examination from Mexico, 4670 participants were followed by telephone calls after being hospitalized because of COVID-19 [20]. In this study, headache and cough were the most frequent symptoms. The authors classified the assessed symptoms into body system clusters but did not carry out hierarchical cluster analysis to show associations like we did.

To better understand the co-prevalence of symptoms and potentially finding keys to potential pathogenic mechanisms of those symptoms and to derive more specific possible treatment approaches long COVID subtypes, cluster analysis has been used by several research groups with long COVID symptoms [6, 8, 21]. The inter-cluster overlap observed in our study suggests that treatment approaches might need to be more holistic, addressing multiple symptom clusters simultaneously.

Our analysis of overlapping clusters also raises questions about the potential risk factors, pathophysiological mechanisms, and treatments underlying these symptom combinations. The use of clustering of long COVID symptoms in treatment can be observed in the development and an occupational therapy intervention. There, only people with fatigue and concentration difficulties were included and the intervention was specified for this target group. In cluster analyses, these symptoms often occur with other neurological symptoms in long COVID patients [22].

Very similar to our approach, a study defined three distinct symptom clusters using hierarchical clustering [9]. Kenny et al. used an identical long COVID definition to our study and included participants with a similar age distribution as in our study. Kenny et al. show fatigue as the most frequent symptom clustered together with pain and musculoskeletal symptoms, similar to cluster A in our analysis. Based on a much larger sample size, our study confirms the cardiorespiratory cluster. Additionally, we found an interesting association of cardiorespiratory symptoms with neurological symptoms such as loss of smell/taste and confusion. Our analysis also shows a more differentiated picture of the symptom clusters, as we not only included the binary appearance of symptoms but also assessed symptom strength.

Our study has several limitations. As our data relies on self-reported information, the assessments and symptoms severity ratings are per se subjective and cannot be verified. Additionally, as participants were self-recruited via the internet, our study might not be representative of the general population. Also, as non-long COVID controls in our study were not inquired on symptom and symptom severity, we could not make comparisons to differentiate clusters. On the other hand, the broad distribution and recruiting of our questionnaire allows to reach participants in regions far away from university medical centers and also to gather data about long-term symptoms after mild COVID-19 courses without the need of hospitalization or intensive care.

When regarding the distribution of clusters within the study population, our clusters are overlapping with symptoms in two or all three clusters occurring in one participant. With the applied method, participants cannot be strictly separated into just belonging to either cluster A, B, or C. Nevertheless, it will be interesting to find out more about risk factors and serological markers associated with the different symptom clusters. In addition, it would be interesting in future research to investigate patient-reported outcomes like the health-related quality of life between the defined clusters.

Having identified certain symptom associations, it could be interesting to link our study results to existing scientific knowledge about post-infectious syndromes and the understanding of their pathophysiology. The COVID Human Genetic Effort consortium recently presented an overview about mechanisms hypothetically explaining key symptoms of long COVID [23]. They postulate that spikes of fever for example (part of our Cluster C) are caused by persistent viral reservoirs while autoimmunity targeting g-protein-coupled receptors on neurons are responsible for neurological symptoms like loss of smell and taste or confusion (as in our Cluster B) [14]. A review and comparison of symptomatology between long COVID and Myalgic Encephalomyelitis/Chronic Fatigue Syndrome (ME/CFS) sheds light on the large overlap of symptoms reported by long COVID studies and the major criteria symptoms for ME/CFS (fatigue, reduced daily activity, and post-exertional malaise) [24]. They suggest a high degree of similarities and discuss the possible origin of both syndromes in a dysregulated immune response and hyper inflammation. As our Cluster A shows a co-prevalence of fatigue with symptoms typically occurring in rheumatologic and auto inflammatory diseases like joint pain, we consider our cluster analysis may give a further piece to the puzzle of distinguishing one or several pathomechanisms of long COVID sequelae.

## Data Availability

Data is available from the corresponding author upon reasonable request.
